# Thalidomide as a potential adjuvant treatment for paraneoplastic pemphigus: A single‐center experience

**DOI:** 10.1111/dth.14353

**Published:** 2020-10-08

**Authors:** Jingying Wang, Yan Zhang, Meng Pan

**Affiliations:** ^1^ Department of Dermatology Rui Jin Hospital, Shanghai Jiao Tong University School of Medicine Shanghai China; ^2^ Department of Dermatology Baoshan Integrated Hospital of Traditional Chinese and Western Medicine Shanghai China

**Keywords:** Castleman disease, paraneoplastic pemphigus, thalidomide

## Abstract

Paraneoplastic pemphigus (PNP) is a rare autoimmune bullous disease associated with an underlying malignancy. The survival rate at 5 years is reported to be as low as 38%. Thalidomide is a medication with strong anti‐inflammatory, immunomodulatory, antiangiogenic, and sedative properties. Recently, the successful application of thalidomide in several dermatological and hematological disorders inspired us to investigate its potential as an adjuvant treatment for PNP. Here, we report our experience of trial thalidomide therapy in 14 PNP patients. After treatment of their associated tumors, the patients were administered thalidomide (75‐100 mg/d) combined with or without low‐ to mid‐dose oral prednisone. Twelve patients completed the therapy. Seven patients (58%) achieved complete remission with no relapse, including two patients who received thalidomide monotherapy. Five patients (42%) died within 1 to 3 months. The 1‐year and 2‐year survival rates in our case series were 58% and 55%, respectively. The regimen was well tolerated. Although the treatment experience presented has a limited sample size and no control, our results imply that thalidomide may be an effective, safe, and economical treatment option for PNP patients. Further research is needed to better understand the mechanisms of action of thalidomide in PNP.

## 1 INTRODUCTION

Paraneoplastic pemphigus (PNP) is a life‐threatening autoimmune bullous disease associated with an underlying malignancy. The clinical symptoms are characterized by painful stomatitis and polymorphous cutaneous lesions mimicking pemphigus vulgaris (PV), pemphigus foliaceus, bullous pemphigoid (BP), erythema multiforme, and lichen planus.^1^ The current first‐line treatment for PNP is high‐dose systemic corticosteroids following tumor removal or control. Other therapeutic options include immunosuppressive adjuvants, plasmapheresis, immunopheresis, intravenous immunoglobulin (IVIg), and biologics such as rituximab. However, none have achieved a satisfactory outcome in regard to improving the poor prognosis. The underlying malignancy, severe infections during immunosuppressive therapy, and bronchiolitis obliterans (BO) lead to relatively high mortality.

Thalidomide is a medication with anti‐inflammatory, immunomodulatory, antiangiogenic, and sedative properties. To date, it has been successfully applied in several dermatological, rheumatological, stomatological, oncological, and hematological disorders.^2^, ^3^ Recent studies inspired us to consider its potential in the treatment of PNP. First, a phase 2 study demonstrated that an oral thalidomide‐cyclophosphamide (CTX)‐prednisolone regimen showed good efficacy and safety in the treatment of multicentric Castleman disease, which is the most frequently associated neoplasm in Chinese PNP patients.^4^, ^5^ The therapeutic effect of thalidomide, probably mediated through its inhibitory action on tumor‐associated cytokines, has also been demonstrated in other solid and hematological malignancies, in which it prevents tumor growth and metastasis. Second, the beneficial effect of thalidomide in the treatment of intractable oral erosions in cases of recurrent aphthous ulceration, Behçet's disease, or lichen planus has raised our interest, as the oral lesions in patients with PNP are also extended, painful, and resistant to treatment. Finally, the sedative effect of thalidomide may help improve sleep quality in PNP patients who suffer from intolerable pain.^2^, ^3^ Thus, we suspect that thalidomide may have multiple advantages as an adjuvant therapeutic modality for PNP. Therefore, we performed a trial of thalidomide therapy in 14 PNP patients and hereby share our preliminary experience.

## 2 MATERIALS AND METHODS

This study was approved by the research ethics committee of Rui Jin Hospital affiliated with the School of Medicine, Shanghai Jiao Tong University (Clinical Ethics Approval 61/2014). Fourteen patients meeting the PNP diagnostic criteria revised by Camisa and Helm^6^ were recruited for our study from October 2010 to December 2019. The patients were informed of the potential benefits and risks of thalidomide and gave written consent for its use.

The clinical findings of the patients are summarized in Table 1. There were nine females and five males with a median age of 40 years (range: 13‐53 years). The median follow‐up time was 32.5 months (range: 1‐91 months).

**TABLE 1:** Summary of the clinical features, treatment regimens, and outcomes of 14 patients with PNP

Case	Age (y) /sex	Time of onset (mo)^a^	Clinical pattern				Underlying neoplasms	With BO	Treatment			Follow‐up duration (mo)	Outcome
			Skin lesions	Mucosal lesions			Location		Surgery	Thalidomide	Other treatment		
				Oral	Ocular	Genital							
1	53/F	2	PV‐like	+	−	−	UCD with follicular dendritic cell hyperplasia	Yes	Yes	100 mg/d Side effect: peripheral neuropathy	Low‐dose prednisone	79	CR
							Retroperitoneal						
2	38/F	9	PV‐like	−	−	−	UCD	Yes	No	100 mg/d	Mid‐dose prednisone, plasmapheresis, IVIg	1	Death due to sepsis
							Mediastinum						
3	20/M	3	EM‐like	+	−	+	FDCS	No	Yes	75 mg/d	Mid‐dose prednisone, IVIg, AZA	55	CR
							Retroperitoneal						
4	13/M	3	EM‐like	+	+	+	UCD	No	Yes	75 mg/d	Mid‐dose prednisone, IVIg	30	CR
							Pelvic						
5	19/F	36	EM‐like	+	−	+	UCD	Yes	Yes	100 mg/d	—	68	CR, with mild respiratory symptoms
							Retroperitoneal						
6	48/M	3	PV‐like	+	+	−	Thymoma	No	No	100 mg/d	Prednisone pulse therapy	1	Death due to sepsis
							Mediastinum						
7	23/F	2	EM‐like	+	+	+	UCD with follicular dendritic cell hyperplasia	No	Yes	100 mg/d	Mid‐dose prednisone, IVIg	54	CR
							Left adrenal region						
8	23/F	2	EM‐like	+	−	−	UCD	No	Yes	75 mg/d	Low‐dose prednisone	35	CR
							Retroperitoneal						
9	47/M	1	PV‐like	+	−	−	Thymoma	No	No	75 mg/d	High‐dose prednisone, IVIg	1	Death due to unknown reasons
							Mediastinum						
10	46/F	5	EM‐like	+	−	−	FDCS	Yes	Yes	75 mg/d	Mid‐dose prednisone, IVIg	3	Death due to BO
							Mediastinum						
11	57/M	17	EM‐like	+	−	−	Thymoma	Yes	Yes	75 mg/d	—	16	CR
							Mediastinum						
12	46/F	2	EM‐like	+	+	−	NHL	Yes	—	100 mg/d	CHOP chemotherapy	2	Death due to lymphoma
13	33/F	6	EM‐like	+	−	−	UCD	Yes	Yes	75 mg/d Side effect: peripheral neuropathy	Mid‐dose prednisone, CTX, IVIg	87	CR, with mild respiratory symptoms
							Left cervical region						
14	42/F	30	PV‐like	+	−	−	UCD	Yes	Yes	50 mg/d Side effect: rash	Mid‐dose prednisone, IVIg	91	PR, persistent stomatitis with mild respiratory symptoms
							Retroperitoneal						
							Retroperitoneal						

Abbreviations: +, involved; −, not involved; AZA, azathioprine; BO, bronchiolitis obliterans; CHOP, cyclophosphamide, doxorubicin, vincristine, and prednisone; CR, complete remission; CTX, cyclophosphamide; EM, erythema multiforme; FDCS, follicular dendritic cell sarcoma; IVIg, intravenous immunoglobulin; NHL, non‐Hodgkin's lymphoma; PNP, paraneoplastic pemphigus; PR, partial remission; PV, pemphigus vulgaris; UCD, unicentric Castleman disease.

^a^ Time of onset referred to the period since the onset of the symptoms till the patient's first visit to our hospital.

Associated neoplasms, such as unicentric Castleman disease (UCD) (n = 8), thymoma (n = 2), follicular dendritic cell sarcoma (n = 2), and non‐Hodgkin's lymphoma (NHL; diffuse large B cell lymphoma) (n = 1), were found in all patients. Ten patients promptly underwent tumor resection, but the other three were not eligible for surgery due to severe respiratory failure or a surgical contraindication. One patient with NHL received cyclophosphamide, doxorubicin, vincristine, and prednisone (CHOP) chemotherapy.

Patients were given oral thalidomide at an initial dosage of 75 to 100 mg/d combined with a glucocorticoid at a low to moderate dosage (oral prednisone, 0.2‐1 mg/kg/d); however, considering their young ages and relatively mild symptoms, patients 5 and 11 received thalidomide monotherapy (initial dosage of 75‐100 mg/d). Additional IVIg, immunosuppressive agents, and plasmapheresis were applied depending on disease severity and treatment response. The dosage of thalidomide was gradually tapered after a satisfactory response was obtained and was maintained at the prolonged consolidation dosage of 25 to 50 mg/d.

Twelve patients completed the therapy. Therapeutic effects were evaluated for both mucocutaneous lesions and systematic disease. Pemphigus disease area index (PDAI) scores were calculated at 0, 6, 12, and 24 months following therapy.

## 3 RESULTS

**FIGURE 1:**
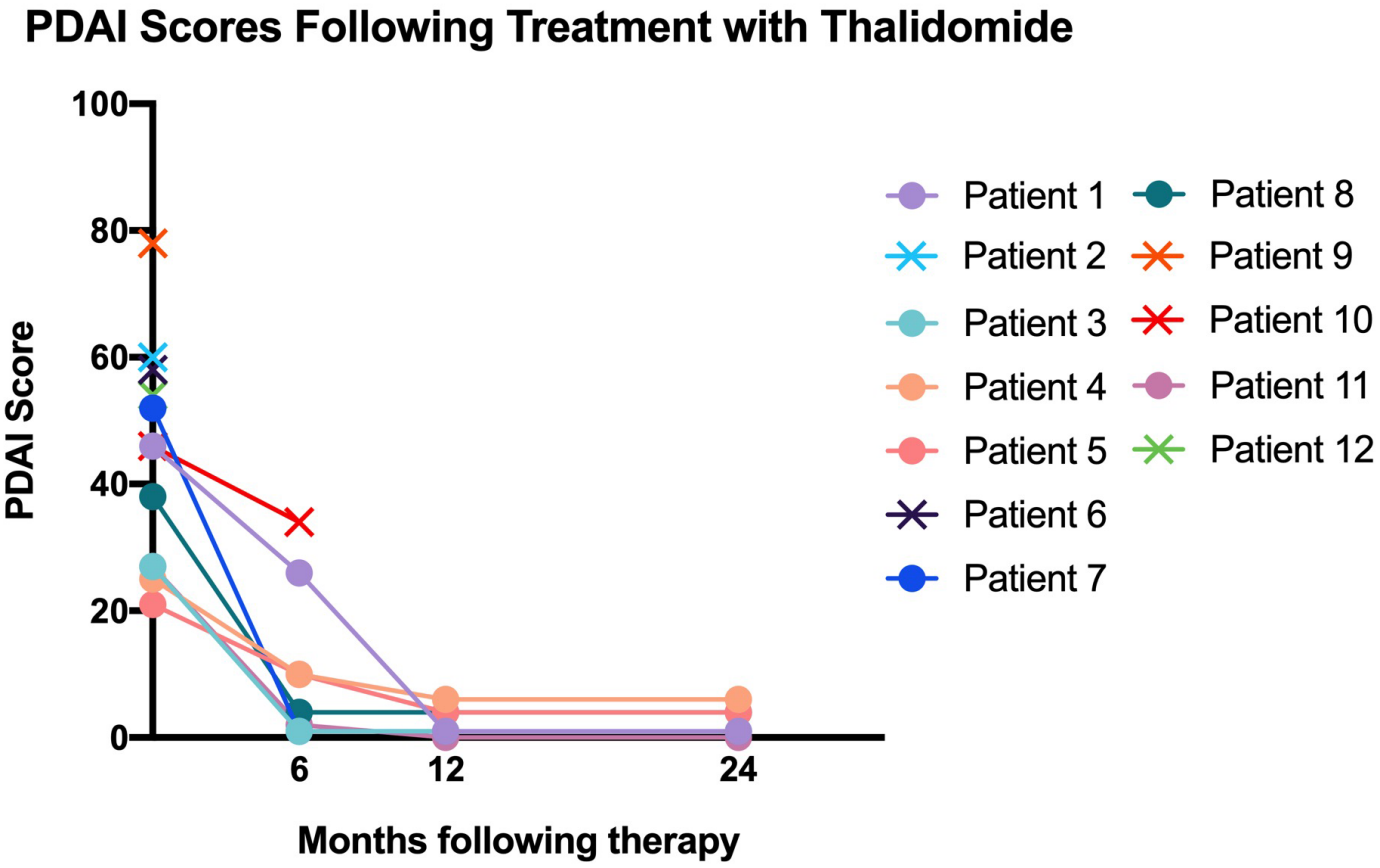
Pemphigus disease area index scores following treatment with thalidomide

**FIGURE 2:**
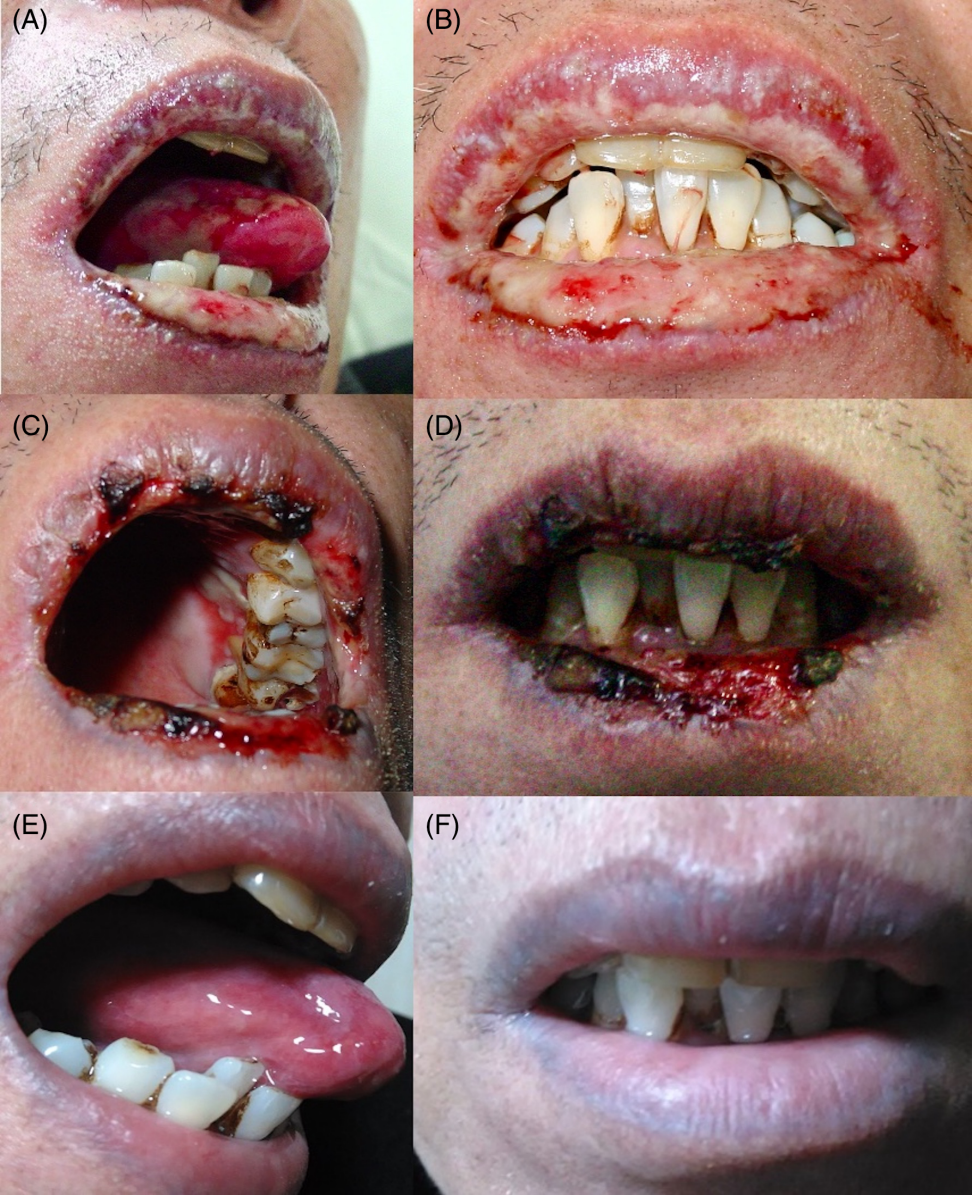
Painful mucosal erosions with effusion in patient 11. (A, B) Significant improvements were seen after 1‐month (C, D) and 6‐month (E, F) courses of thalidomide monotherapy

**FIGURE 3:**
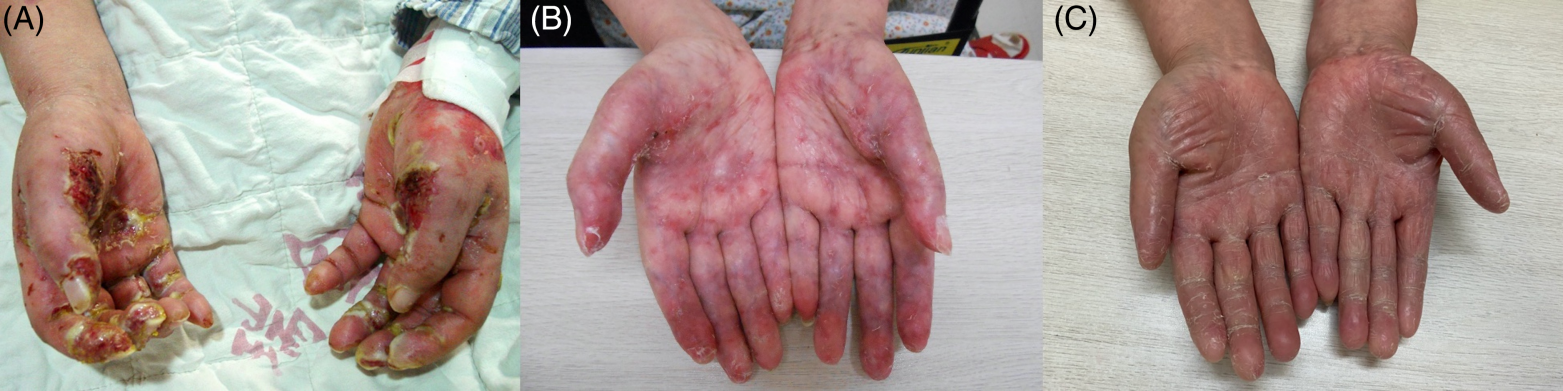
Severe pemphigus vulgaris‐like lesions on the hand of a patient before treatment. (A) Lesions were significantly relieved under thalidomide therapy combined with low‐dose prednisone for 3 (B) and 6 months (C)

The PDAI scores of the patients showed a trend indicating improvement during the follow‐up period (Figure 1). The treatment duration required for mucocutaneous remission varied from 7 to 18 months (Figures 2 and 3). Skin lesions achieved complete remission or significant improvement in 3 months, while for mucosa damage, more than half a year was needed. Eight patients (57%) developed BO 1 to 6 months after the onset of disease. Seven patients (58%), including the patients who received thalidomide monotherapy, achieved complete remission of their skin lesions and tumors under the therapy. Five patients (42%) died within 1 to 3 months due to aggressive respiratory failure (n = 1), sepsis (n = 2), lymphoma (n = 1), or unknown reasons (n = 1). Notably, these five patients included four who did not undergo surgery, which highlighted the paramount importance of the management of the underlying tumor. The 1‐year and 2‐year survival rates in our case series were 58% and 55%, respectively. The mortality rate of the patients with benign neoplasms was 33%, which was much lower than the rate of 66% for the patients with malignancies. For the subjects who survived, five with BO were well controlled by inhaled bronchodilators and corticoids, with remaining mild or no respiratory trouble. All these patients showed no recurrence under close surveillance and reached the end of the follow‐up period with maintenance with a minimum dosage of thalidomide (<50 mg/d) and/or prednisone (<10 mg/d). One patient was able to go off the medication and experienced no relapse during the 2‐year follow‐up period (patient 7).

On the other hand, we observed good tolerance of thalidomide in most patients. Three patients experienced slight adverse effects during the treatment, including numbness of limbs (n = 2, patients 1 and 13) and facial erythema (n = 1, patient 14). Symptoms were spontaneously relieved after thalidomide therapy was discontinued. Patient 1 restarted thalidomide treatment without any discomfort after a pause of several months. The others were prescribed alternating CTX and IVIg. Patient 13 responded well to the therapy, while patient 14 demonstrated relatively stable disease except for persistent oral erosions.

## 4 DISCUSSION

Although the pathogenesis of PNP remains unknown, it seems plausible that both antibody‐mediated immunity and cell‐mediated immunity play key roles. One hypothesis is that tumor antigens can act as a trigger of cell‐mediated interface dermatitis and subsequent progressive humoral autoimmunity. The epitope‐spreading phenomenon gives rise to the presence of various autoantibodies, including antibodies against plakin family proteins, desmocollins, desmogleins, and alpha 2‐macroglobulin‐like protein 1.^7^, ^8^

Therefore, treatment of PNP has two goals: (a) removal or control of the underlying tumor and (b) suppression of dysregulated autoimmunity. For patients with benign underlying solid tumors, surgery alone can sometimes produce large improvements by decreasing circulating autoantibody levels. However, refractory stomatitis and systematic complications may still be present in most postoperative patients, as well as those who cannot undergo surgery, who respond poorly to the conventional therapy of high‐dose corticosteroids.^9^

Prior use of thalidomide in pemphigus and pemphigoid groups, namely, PV, BP, cicatricial pemphigoid, and Hailey‐Hailey pemphigus, has been reported to be effective.^10^ Three sporadic previous cases of PNP with UCD were also treated with thalidomide as part of the immunomodulatory therapy applied. Two of these patients obtained satisfactory outcomes, while one died from respiratory failure.^11^, ^12^, ^13^

Our study is the first case series report concerning thalidomide in the treatment of PNP. We preliminarily achieved favorable results, producing disease control, a reduced mortality rate, and few adverse effects. A multicenter retrospective cohort study in France reported 1‐year, 2‐year, and 5‐year survival rates of 49%, 41%, and 38%, respectively.^14^ Another single‐center cohort study from China reported much higher survival rates in the PNP patient group with Castleman disease, which were 76.9% and 57.6% at 1 and 3 years, respectively. These patients received standard treatment with IVIg and corticosteroids in combination with surgical tumor removal.^15^ This difference may result from a different spectrum of underlying diseases. In our series, patients with UCD had a more favorable prognosis, especially those who underwent surgery, which is consistent with previous studies. All postoperative patients with UCD receiving thalidomide therapy with or without relatively low‐level corticosteroids achieved stable symptom‐free remission, except for mild respiratory problems (follow‐up period ranging from 30 to 79 months). Therefore, we speculate that thalidomide improves the prognosis and quality of life of PNP patients, especially those with Castleman disease who undergo surgery.

In our series, the incidence of BO was 57% and the mortality due to respiratory failure among these patients was 12.5%, which was similar to the data reported in another Chinese PNP series.^16^ However, another American study of PNP patients with Castleman disease showed that BO developed in 26 of 28 patients and caused fatal outcome due to respiratory failure in 22 patients.^11^ It can be seen that in Eastern and Western countries, not only the commonly underlying tumors of PNP are heterogeneous, but also the incidence and outcome of BO seem to be different. Therefore, further multicenter research is needed to investigate the distinct disease pattern.

Our study had some limitations in terms of the small sample size, single‐center design, and lack of a control group. However, due to the fatal prognosis of PNP, given humanitarian considerations, rigorous design of clinical trials is challenging to implement. Despite these limitations, we believe that thalidomide adjuvant therapy has prospects for further application and research.

Although the mechanism is still elusive, previous studies have suggested that thalidomide exerts strong anti‐inflammatory and immunomodulatory effects by inhibiting various cytokines, including tumor necrosis factor‐α, vascular endothelial growth factor, interleukin (IL)‐6, IL‐1, IL‐12, and IL‐10, and interferon‐γ, and possibly nuclear factor‐кB.^2^, ^3^ Among these cytokines, IL‐6 and IL‐10 were reported to be present at elevated levels in PNP patient serum.^17^, ^18^ In addition, for associated tumors, thalidomide may downregulate the expression of vascular endothelial growth factor, which is essential for tumor growth and dissemination.^3^ IL‐6 is also believed to play an essential role in Castleman disease.^4^ Together, these findings may establish a theoretical basis for including thalidomide in the treatment of PNP.

Thus, we recommend a therapeutic regimen based on the application of thalidomide with low‐ to mid‐dose prednisone. Although the presented work is a small‐sample treatment experience, our observations imply that thalidomide could be an effective, safe, and economical treatment option for PNP patients. Further research is needed to confirm this conclusion and better understand the mechanisms of action of thalidomide in PNP.

## CONFLICT OF INTEREST

The authors declare no potential conflict of interest.

## Data Availability

The data that support the findings of this study are available from the corresponding author upon reasonable request.
